# Assessing health, wellbeing, and working life impacts of a 4-day week: a multimodal protocol

**DOI:** 10.3389/fpsyg.2026.1894642

**Published:** 2026-08-17

**Authors:** Rhiannon M. Armitage, Nicholas E. Souter, Tessa J. Graftdijk, Joanna McLaren, Sinéad A. Moore, Christina Kampoureli, Chris Racey, Daniel Campbell-Meiklejohn, Sarah N. Garfinkel, Lisa Mullen, Emma Russell, Charlotte L. Rae

**Affiliations:** 1 School of Psychology, University of Sussex, Brighton, United Kingdom; 2 Department of Neuroimaging, Institute of Psychology, Psychiatry & Neuroscience, King's College London, London, United Kingdom; 3 Sussex Neuroscience, University of Sussex, Brighton, United Kingdom; 4 Institute of Cognitive Neuroscience, University College London, London, United Kingdom; 5 Department of Clinical & Experimental Medicine, Brighton & Sussex Medical School, Brighton, United Kingdom; 6 Business School, University of Sussex, Brighton, United Kingdom

**Keywords:** 4-day week, health, wellbeing, work performance, working time reduction

## Abstract

Long working hours can negatively affect workers' health, wellbeing, and work performance. Previous trials reducing working time have found improvements in multiple aspects of wellbeing, including sleep, job satisfaction, and reduced burnout, while maintaining performance. However, the potential psychological and biological mechanisms underlying these improvements, including immune function and brain function, remain unexplored. Therefore, the aim of this study is to understand the bidirectional relationship between working patterns and physical and mental health, and how workplace performance is affected. We aim to test at least 150 working-age adults in the UK (approximately half with a working time reduction and no loss of salary, and half controls with no change in working patterns) in a 14-week trial, including a 2-week baseline period, with additional participants completing questionnaires only. The primary outcome measure is the difference in physical and mental health from baseline to the end of the trial among participants undergoing a reduction in working time, compared with the control group. Data collection is currently underway, and while the majority of employers in the working time reduction group adopt a 4-day week format, the specific change in working patterns is flexible and varies between employers according to their operational need, including a 9-day fortnight, two half-days and shorter working days. Core variables include brain structure and function, sleep, immune function, interoception (perception of bodily sensations), time use, diet, mental health, and workplace performance and engagement. Secondary outcomes include weekly monitoring of self-reported wellbeing changes to identify key inflection points and assess whether these are maintained at a 6-month follow-up. Overall, this study aims to generate highly novel multimodal data on the psychological and biological implications of working time reduction, including observed changes in health, wellbeing, and performance.

## 1 Introduction

Aspects of working life, including the amount of time spent at work, can negatively impact health and wellbeing (Afonso et al., 2017). In addition to affecting employees' quality of life, adverse health outcomes reduce performance and economic output by increasing the number of lost workdays (Bryan et al., 2021; Martinez et al., 2025). One approach to mitigate these harmful effects is to reduce working time. Under working time reduction models, employees' work time is reduced without a corresponding reduction in pay. In this way, working time reduction is distinct from part-time work, in which pay is reduced in proportion to working hours, and from compressed-hours contracts, whereby pay is maintained but the same working hours are compressed into fewer days rather than reduced. Under a 4-day week, for example, full-time employees typically transition from 5 to 4 days of work with no change in pay. The 4-day week has been described as a “100–80–100” working time reduction model, meaning that 100% of previous performance is undertaken in 80% of previous working hours, whilst retaining 100% of previous salary (Barnes and Jones, 2020). While the 4-day week is perhaps currently the most common form of working time reduction, other arrangements may be used, including a 9-day fortnight (one full workday reduced every other week), two half-days per week, or shorter days (Fan et al., 2025).

Landmark research on the effects of working time reduction has provided critical insight into the effects on employee wellbeing. Fan et al. (2025) present evidence from national cohort trials coordinated by the non-governmental organization 4 Day Week Global, drawing on data from the UK, Ireland, Australia, New Zealand, Canada, and the USA. Self-report data were collected from employees undergoing working time reduction (*n* = 2,896) and control employees experiencing no change in working time (*n* = 285). Following working time reduction, participants reported improvements in burnout and job satisfaction, suggesting benefits to employees' subjective experience of work. Additionally, participants reported improvements in both mental and physical health. These changes were not observed in control participants. Benefits were dose-dependent: greater individual reductions in working hours predicted greater improvements in wellbeing. Furthermore, improvements in wellbeing were mediated by reported changes in sleep problems, fatigue, and perceived work ability. Another working time reduction trial in Sweden also noted improvements in self-reported sleep quality and duration (Schiller et al., 2017). Indeed, self-reported improvements in wellbeing following the implementation of working time reduction are evident across multiple geographical contexts, including the UK (Lewis et al., 2023), Germany (Backman et al., 2026; Backmann et al., 2024), Portugal (Gomes and Fontinha, 2024), Iceland (Haraldsson and Kellam, 2021), Sweden and Norway (Lid Falkman et al., 2026). A recent scoping review of working time reduction trials reported consistent benefits to wellbeing, spanning physical health, mental health, and work-life balance (Utzet et al., 2026).

The findings from these working time reduction trials are supported by research exploring the relationship between length of working week, working patterns and aspects of health and wellbeing. Afonso et al. (2017) observed that participants working more than 48 h per week experienced greater symptoms of anxiety and depression, and poorer sleep quality, compared to those working fewer hours. Associations between longer working hours and sleep and mental health have also been noted in many other investigations (Nakashima et al., 2011; Ogawa et al., 2018; Ropponen et al., 2018; Virtanen et al., 2011, 2018; Virtanen and Kivimäki, 2012; Weston et al., 2019, although see for conflicting results: Varma et al., 2012). In addition, working 55 or more hours per week has been associated with less healthy diets and lower fruit intake (Baek et al., 2024). In a Korean all-female sample, Kwak et al. (2019) observed that shift workers had higher C-reactive protein levels, which are associated with poorer immune responses, than daytime workers. Longer working hours have also been associated with poorer physical health, with those working longer hours demonstrating a higher incidence of periodontitis, an advanced gum disease (Lee et al., 2021), and coronary heart disease and stroke (Kivimäki et al., 2015; Virtanen et al., 2012).

One might intuitively expect any reduction in working time to result in a proportional decline in performance. Performance refers generally to the observable quality of an individual's work. Within this, “productivity” is reflective of quantifiable outputs derived from one's performance, such the tasks achieved (Rae and Russell, 2025). Evidence in fact suggests that work performance is maintained, or sometimes even improved, when working time reduction models are applied. Across companies participating in the 2022 UK national trial, for instance, revenue remained stable, and staff turnover fell by 57% after implementing working time reduction (Lewis et al., 2023). At a more local level, a public sector trial in one English Council, found that of 24 key performance indicators (KPIs), 22 were maintained or improved, and staff turnover was reduced by 39% (Ainsworth, 2024). This was calculated to amount to a full year net cost saving of £371,500. Similarly, most business leaders participating in the Portuguese trial reported increased revenue relative to the previous year, alongside reductions in absenteeism and staff turnover (Gomes and Fontinha, 2024). The 2024 German working time reduction national trial showed no significant change (increase or decrease) in profit or revenue (Backmann et al., 2024), and performance was similarly found to be stable following a working time reduction trial conducted in Scotland's public sector (The Autonomy Institute, 2025). This maintenance of performance levels was attributed to work reorganization changes, including the implementation of “focus periods” for work, increased meeting efficiency, and the automation of common tasks.

The exact mechanisms by which reducing working time may benefit wellbeing without compromising performance remain unclear. However, observed benefits may be partly explained by the mitigation of detrimental health effects of long working hours. The Conservation of Resources (COR) theory (Hobfoll et al., 2018) suggests that workers are motivated to obtain and retain things they value. When resources that facilitate this (e.g., energy or time) are consistently stretched or depleted, an individual enters a protective state in which effective work is less likely, and the likelihood of burnout increases (Hobfoll, 1989; Hobfoll et al., 2018). The Job Demands-Resources (JD-R) model similarly considers the interplay between job demands, elements of work that require mental or physical effort, and job resources, factors that enable workers to achieve goals and personally develop (Bakker and Demerouti, 2007; Demerouti et al., 2001). When job demands exceed the resources available to deal with these, this can lead to job strain, which may undermine both health and work performance (Schaufeli et al., 2009). Meanwhile, Sonnentag et al. (2017) emphasize that resources depleted at work need to be restored through engagement in recovery processes, such as psychological detachment from work (Sonnentag and Fritz, 2015). Insufficient recovery experiences can impact wellbeing (Fritz et al., 2010; Sonnentag et al., 2010). Vandoros et al. (2025) highlight that transitions to reduced working time may empower employees to work more efficiently and adopt better time-management strategies, while health benefits stem from increased time allocated to recovery behaviors. Rae and Russell (2025) propose that observed benefits of working time reduction may operate through two complementary psychological mechanisms, with individuals being (a) better rested and (b) more motivated at work, both of which build resources to help manage job demands, and are facilitated by adaptive changes in brain function.

Indeed, emerging evidence links occupational stress to changes in brain structure and function. Neuroimaging studies have associated chronic work stress with cortical thinning of the frontal cortex (Blix et al., 2013; Savic, 2015), and localized enlargement of the amygdalae (Nasrullah et al., 2023), changes which have been associated with executive dysfunction (Yuan and Raz, 2014) and mental health conditions (Li et al., 2025), respectively. Conversely, Jang et al. (2025) observed that participants working more than 52 h per week had greater volume in frontal regions than those working fewer than 52 h. Functional disruptions have also been reported; participants experiencing greater occupational stress demonstrated altered prefrontal activity during verbal fluency tasks (Chou et al., 2016; Kawasaki et al., 2015), and reduced connectivity between the amygdala and both the dorsolateral prefrontal cortex and the anterior cingulate cortex, in conjunction with impaired emotion regulation (Golkar et al., 2014). A recent study using data from the UK Biobank found that participants working longer hours exhibited greater within-network connectivity in the somatomotor network (Souter et al., 2026). This association may reflect a maladaptive response in the context of highly demanding work, or an adaptive improvement to one's ability to work long hours; research using an intervention design would be required for further insight. To date, however, the effects of working time reduction on brain function using an intervention design have not been directly assessed.

Despite interesting wellbeing results from previous working time reduction trials, many share key methodological limitations. A review of working time reduction academic research by Campbell (2024) highlights the possibility of the Hawthorne effect (whereby participants' responses are influenced by their affinity for the research topic and motivation to report socially desirable outcomes), as well as a focus on self-report measures. Other limitations previously highlighted include a lack of data on individual compliance with working time reduction transitions, small and unrepresentative samples, and insufficient control groups (Cuello, 2023). Together, these limitations create opportunities for working time reduction study protocols that enhance scientific rigor and the potential to translate findings into policy change.

The project outlined below aims to build on the above findings, address identified limitations, and elucidate the mechanisms by which reducing working time simultaneously benefits performance and wellbeing. The “Sussex 4-Day Week” trial examines how working time reduction relates to outcomes across multiple health domains, including brain function (using functional magnetic resonance imaging), inflammatory cytokines (using blood serum samples), interoception (using heartbeat perception tasks), sleep (using actigraphy sleep watches and diaries), time use (using self-report diaries), and aspects of mental health and experience of work life (using self-report questionnaires). The inclusion of objective biomedical data addresses a key limitation of many historical trials, which often rely solely or primarily on self-report data. Additionally, this project aims to include a balanced control group not undergoing working time reduction to elucidate causal mechanisms underlying any observed effects. This protocol paper outlines the methodological framework for this investigation. To our knowledge, this is the first study to use a comprehensive multimodal protocol that integrates self-report measures, behavioral tasks with physiological monitoring, actigraphy sleep data, blood markers of inflammation, and functional neuroimaging to assess the effects of reduced working hours on the mind, brain, and body.

### 1.1 Hypotheses

Across the multimodal protocol, we formulated hypotheses for each of our measures based on the existing literature and the proposed mechanistic pathways. These hypotheses specify the expected effects of working time reduction across the key behavioral, physiological, and neurobiological outcomes listed above.

#### 1.1.1 Questionnaires

We record weekly measures of indicators of wellbeing, and performance to test how rapidly these change over time, informed by literature on recovery processes (Sonnentag and Fritz, 2015), COR theory (Hobfoll et al., 2018), and JD-R model (Bakker and Demerouti, 2007). We collected baseline and end-of-trial measures on additional occupational, wellbeing, and lifestyle measures commonly examined in workplace wellbeing research and that reflect longer-term resource changes as described in COR theory, JD-R model, and research on recovery processes (Bakker and Demerouti, 2007; Hobfoll, 1989; Sonnentag and Fritz, 2015). Based on these frameworks and previous findings from working time reduction research, we expect overall improvements in employee wellbeing and performance measures following working time reduction, compared to the control group.

#### 1.1.2 Time-use diaries

To understand how time use changes with reduced working hours, participants can opt in to complete a time-use diary at the start and end of trial. Self-report paper time-use diaries have corresponded closely with objective measures of daily activities (Gershuny et al., 2020). Based on previous working time reduction studies, we expect employees in the 4-day week group to reallocate their time following the trial, with changes in activity categories such as travel time, household work, and free-time activities (Mullens et al., 2021; Schiller et al., 2018).

#### 1.1.3 Sleep watch and diaries

Poor sleep is strongly associated with work-related stress and has significant consequences for workplace performance (Krause et al., 2017; Querstret and Cropley, 2012; Schiller et al., 2017; Wolkow et al., 2016; Yao and Basner, 2019). Sleep quality may be further compromised by work-related nighttime rumination, whereby employees continue to cognitively engage with work during non-work hours. In contrast, psychological detachment, the ability to mentally disengage from work during leisure time (Sonnentag and Fritz, 2007), is protective against these detrimental effects (Querstret and Cropley, 2012) and may be enhanced under a 4-day working week. Previous working time reduction studies, involving shorter daily working hours, suggest improvements in sleep quality and duration (Åkerstedt et al., 2001). However, these studies have not typically employed research-grade actigraphy (i.e., wearable sleep monitors) or examined finer-grained sleep metrics, and such objective sleep assessment has not yet been applied within a 4-day week trial. We expect improvements in sleep duration and quality during the trial for those undergoing a working time reduction. These effects may be enhanced by increased psychological detachment.

#### 1.1.4 Blood sample

To evaluate immune function and inflammation, a blood sample is collected at the start and end of the trial to measure the serum concentrations of prototypical inflammatory markers, including C-reactive protein (CRP), and pro-inflammatory cytokines such as, interleukin-1 beta (IL-1β), interleukin 6 (IL-6), and tumor necrosis factor alpha (TNF). The serum concentrations of these molecules are known to be affected by stress, sleep, and poor mental health (De Azevedo Cardoso et al., 2023; Kennedy and Niedzwiedz, 2022; Reinhardt et al., 2016; Tang et al., 2025; Wolkow et al., 2015), but have not been investigated in relation to working time reduction. We expect a decrease in pro-inflammatory cytokine and CRP expression after a reduction in working time, likely in relation to improvements in other wellbeing markers, such as lower stress levels and improved sleep.

#### 1.1.5 Interoception measures

Interoception is the sensing, interpretation and integration of signals originating from within the body by the nervous system, providing conscious and unconscious mapping of the body's internal systems on a moment-by-moment basis (Khalsa et al., 2018). Multi-dimensional models of interoception distinguish multiple dimensions of processing, including *interoceptive accuracy*—correct and precise monitoring of performance on objective behavioral tests of interoception; *self-report and interoceptive beliefs*—self-evaluated assessment of beliefs concerning interoceptive sensations and experiences accessible consciously and sub-consciously; and *interoceptive insight*—metacognitive awareness of experience and performance, e.g., confidence-accuracy correspondence (Suksasilp and Garfinkel, 2022). Furthermore, *trait interoceptive prediction error* assesses the discrepancy between objective accuracy and self-report interoceptive beliefs (Garfinkel et al., 2016). Greater positive prediction error (e.g., lower accuracy, higher self-report and interoceptive beliefs) is associated with anxiety (Garfinkel et al., 2016).

We expect an improvement in interoceptive accuracy following reduction in working hours, as reduced stress and better sleep are associated with enhanced interoceptive accuracy (Ewing et al., 2017). Furthermore, in clinical samples across various psychiatric diagnoses, lower accuracy is often accompanied by greater interoceptive beliefs than in control samples (Critchley et al., 2023; Garfinkel et al., 2016; Rae et al., 2019). Therefore, after working time reduction, lower stress and better mental health over a period of several weeks may be associated with a reduction in interoceptive beliefs, through less intense and/or less frequent self-perceived sensations, as assessed by self-report questionnaire (Body Perception Questionnaire above). Collectively, this may reduce trait interoceptive prediction error.

#### 1.1.6 MRI scan

Multiple magnetic resonance imaging (MRI) sequences were chosen to investigate specific aspects of brain organization and function relevant to working time reduction. These include three functional MRI (fMRI) scans; two task-based scans investigating response inhibition and interoceptive focus; resting-state, diffusion-weighted imaging, and T1- and T2-weighted structural scans. Firstly, a stop signal task tests neural processes underlying response inhibition. This will measure changes in prefrontal function (e.g., Korucuoglu et al., 2021; Sharp et al., 2010). Second, an interoceptive focus task tests neural processes of interoceptive attention (Avery et al., 2015; Livermore et al., 2024)—the purposeful observation of internal bodily signals (Suksasilp and Garfinkel, 2022). This will measure changes in regions that process bodily stress signals, including the insular cortex (e.g., Craig, 2002; Schulz, 2016; Tan et al., 2018; Wiebking et al., 2015). Thirdly, resting-state fMRI will assess how signals within and across regions and networks covary with each other in the absence of explicit task instructions. From this, connectivity measures are extracted for regions and networks of interest. For example, connectivity in the default mode network is sensitive to stress (Clemens et al., 2017) as well as fatigue and depression (Høgestøl et al., 2019). Effects may also manifest as alterations in the differentiation of unimodal and multimodal regions, as measured using cortical gradients (Bernhardt et al., 2022; Margulies et al., 2016). Finally, diffusion-weighted MRI reflects white matter structure. Elements of lifestyle (sleep, diet, physical activity) are associated with white matter structure (Bruce et al., 2023; Li et al., 2020; Voldsbekk et al., 2021). As we expect lifestyle factors such as sleep, diet, and physical activity may change with working time reduction, it is possible such lifestyle changes are associated with changes to white matter.

## 2 Methods and materials

### 2.1 Participants and inclusion criteria

The study protocol was approved by the Brighton and Sussex Medical School Research Governance and Ethics Committee (RGEC; protocols ER/BSMS6337/4 and ER/BSMS6337/6) and conducted in accordance with the ethical standards set by the British Psychological Society and the Declaration of Helsinki. The study recruits two groups of participants: those undergoing working time reduction and control participants who do not change working hours.

We aim to recruit a balanced sample of at least 150 participants for all measures (with approximately half who reduce their working time and half controls). Sample size was determined using a power calculation in G^*^Power on questionnaire pilot data (*n* = 30) with an effect size of 0.45, alpha of 0.05 and power of 0.95. This suggests a group sample size of at least 68 is required. Furthermore, participation in certain study components is voluntary (e.g., MRI, blood sample, interoception, sleep, and time-use diaries), and therefore sample sizes are expected to vary across modalities as a consequence of participant self-selection and differential uptake of specific measures; accordingly, statistical power is also expected to vary between outcome domains. However, as this is the first working time reduction trial to use non-self-report measures, it was not possible to estimate power calculations beyond questionnaire data. In addition, as we will not examine moderating subgroup effects within the control group, fewer controls may be required. The primary purpose of the control group is to account for factors unrelated to the intervention itself, including practice effects, familiarity with the MRI scanning environment, and temporal changes that may occur irrespective of working time reduction. This will allow us to determine whether observed changes in outcomes can be attributed to the working time reduction intervention rather than repeated testing or assessment effects.

Participants in the working time reduction group are expected to achieve a “meaningful reduction” in average weekly working time, with original pay maintained (on par with inclusion criteria of recent national cohort trials, e.g., Fan et al., 2025). The majority of participants have switched to a 4-day week, with one full workday reduced each week. However, participants are not required to implement a 4-day schedule, and a minority have adopted other forms of work-time reduction, including a 9-day fortnight with one full workday reduced every other week, two half-days per week, or shorter days. This aligns with recent international cohort trials, in which the majority of participants switched to a 4-day week but were permitted to adopt other schedules according to organizational and individual preferences (Fan et al., 2025). For participants in this group, the research programme is explicitly referred to in recruitment materials as a “*4-day week study*.” Given the heterogeneity of work patterns adopted by employers across the trial, participants undergoing working time reduction will be referred to as the “working time reduction group” throughout this paper.

Control participants do not undergo any reduction in working time between the start and end of the research period. They maintain their original work schedule and pay. For participants in this group, the programme is referred to in recruitment materials as a “*workplace wellbeing study*.” No active deception is used; participants in this group are aware they are part of a larger study in which another group is receiving a reduction in working time.

### 2.2 Protocol optionality

Participation in the questionnaire aspect of the programme is offered to all staff in participating organizations, regardless of their original working schedule (e.g., part-time status, compressed hours, etc.). However, participation in any additional remote (see Section 2.9) or in-person measures (see Section 2.10) is offered only to those working full-time at the start of the study period, due to limited resources for acquiring specific types of data (e.g., MRI scanning costs, sleep-watch availability). Therefore, everyone, regardless of working pattern, completes the questionnaires, and full-time staff have the option to volunteer for additional remote or in-person measures

### 2.3 Recruitment

Employers have been recruited on a rolling basis between 2022 and 2026. Unlike most national cohort-based working-time-reduction trials, this protocol requires local recruitment because several core measures, including MRI scans, blood samples, and interoception measures, must be conducted in person at the University of Sussex. As only a minority of staff at each organization volunteer for in-person testing, a large pool of local employers is needed to achieve adequate sample sizes on these measures. Therefore, we prioritize organizations based near the research site, and invite them to join the trial at a time that suits their operational needs. Recruitment for both control and working time reduction participants is supported through local business newsletters, community talks by the senior researcher (Rae), public engagement events, research participation bulletins, social media posts, and word of mouth. A small number of employers participating in UK national pilots coordinated by the 4 Day Week Foundation have also opted into the protocol, alongside the national programmes.

Employers are primarily contacted through a senior decision-maker (e.g., CEO, Managing Director, or Human Resources lead), who determines whether their staff will implement working-time reduction or serve as controls. Except for one organization that contributed to both groups, all employees within a given organization are assigned to the same group. Organization leaders inform staff about the study and, where applicable, the planned reduction in working time. Staff are invited to a discovery call with the senior researcher, after which they can opt into the study and select which components they wish to complete (i.e., questionnaires only, additional remote measures, or an in-person visit). Participation in the research is voluntary and independent of eligibility to take part in time management training (see Section 2.5) and, for the working time reduction group, participating in their employer's working time reduction trial.

Most participating organizations are small- or medium-sized enterprises or micro businesses, consistent with other recent working-time-reduction trials (e.g., Fan et al., 2025). A small number of participants take part independently, rather than being recruited as part of an employer, including self-employed individuals. Control organizations are intentionally matched on size and sector, although precise pair-matching is not feasible due to geographical constraints. As recruitment relies on proximity to the research site, the final sample will represent a convenience sample rather than a stratified one. However, a range of sectors are represented in both groups, which will be fully reported in the respective data analysis papers.

### 2.4 Compensation

Participants in both the working time reduction and control groups are equally compensated for their time. All participants complete the self-report questionnaires and are offered £50 for their participation (with a minimum completion threshold of 80% of questionnaires for reimbursement). Missed questionnaires are excused in cases of annual leave or sick leave for which we have been informed. Each additional measure is reimbursed separately (£10 for sleep watch and/or time-use diary, £5 for blood sample, £10 for interoception measures, £25 for MRI).

As an additional incentive, we provide anonymised company-level reports to the business with summary results from the wellbeing and workplace questionnaires: baseline vs. end of trial, as well as changes across the weeks for their weekly questionnaires (see Section 2.8 for a summary of these measures). We also offer personalized reports for individual participants on their data from the same measures. For those who undergo an MRI brain scan, we provide an image of their brain. As an incentive for additional time completing the 6-month follow-up, companies and/or individuals can receive an updated report with 6-month follow-up data. No additional participant reimbursement is given at 6-month follow-up.

### 2.5 Time management training

Organizations undertaking working time reduction typically undergo work reorganization to accommodate the shorter working week and maintain or improve performance (Fan et al., 2025; Pang, 2023). As part of the research programme, participants are offered a time management training session during the baseline period (before working time reduction commences).

Our team designed this training to mirror that offered to organizations in recent cohort working time reduction trials. The 4 Day Week Global trials offered webinars to help organizations undergo work reorganization, in which low- and zero-value activities, such as unnecessary meetings, are reduced (Fan et al., 2025). The training in the present protocol is a 1-h focus group activity, in which participants receive a short presentation on time management strategies, such as task prioritization, meeting etiquette, and messaging habits. Staff are then asked to reflect on their current time use, identify areas of inefficiency, and develop strategies for work reorganization to use during the trial.

The session is led by the senior researcher (Rae) and delivered online or in person, according to the organisation's preference. For larger companies, multiple sessions are hosted to accommodate more participants while keeping individual session sizes conducive to a workshop (maximum eight participants per session). We offer time management training to both working time reduction and control groups, enabling investigation of the unique effects of working time reduction (which may include increased motivation to use time-management strategies and reorganize work), beyond merely receiving time-management advice.

### 2.6 Data acquisition and protocol overview

**Figure 1:**
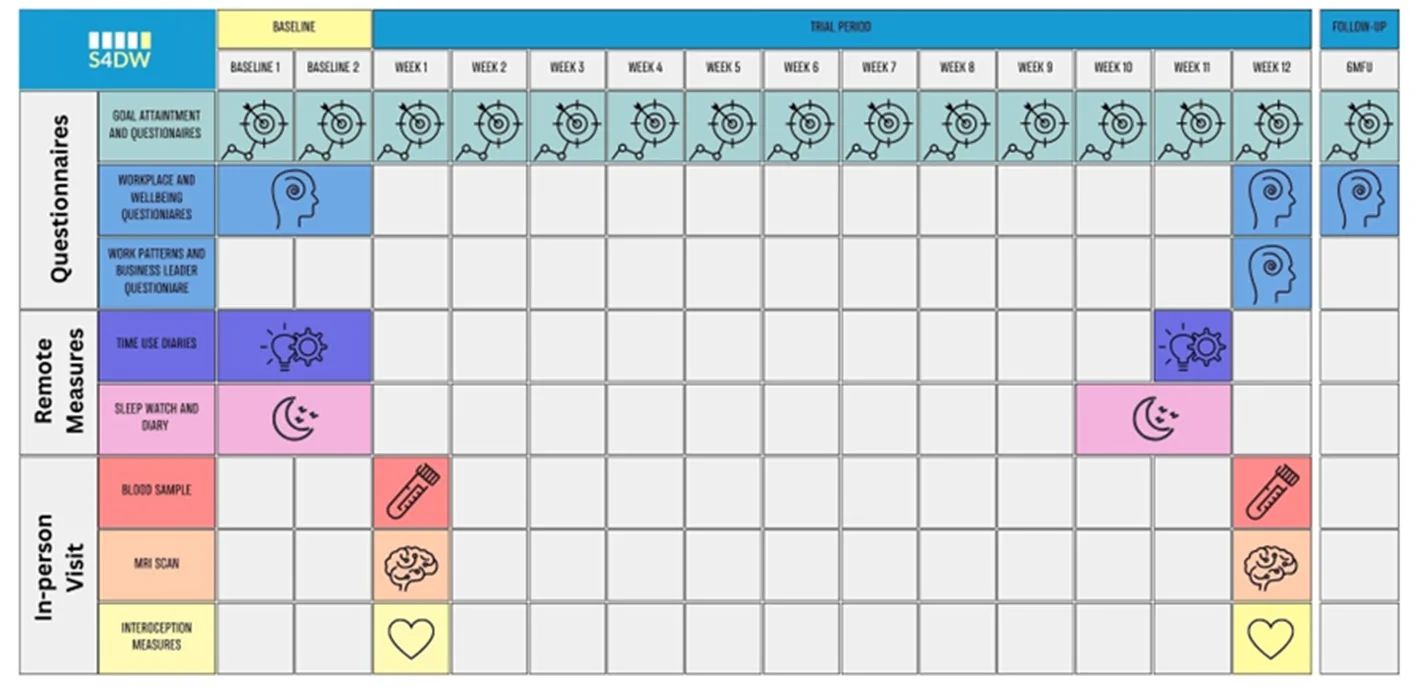
The full protocol timeline for both the working time reduction intervention and control groups.

The research protocol runs for 14 weeks, during which participants complete weekly questionnaires. In the first two baseline weeks, participants maintain their existing working patterns. At baseline, all participants complete wellbeing and workplace questionnaires, and additional remote or in-person measures if chosen. Over the subsequent 12 weeks, participants in the working time reduction group implement their planned reduction in working time, while those in the control group do not change working time. During the final 3 weeks, initial assessments conducted during the baseline period are repeated, alongside additional work patterns and questionnaires for business leaders. We also include an optional 6-month follow-up that repeats the baseline questionnaires. A summary of the protocol is shown in Figure 1. The section below provides details of all assessments used.

There have been two waves of data collection. An initial project to investigate the biological markers of working time reduction was initiated in 2022, with institutional funding. Subsequently, external grant funding supported a second wave of data collection (with no hiatus between the two waves). Data collection has been underway since March 2022, with control employers from 2025, except for a single control employer in 2022. We anticipate completing data collection in 2027. During this time, there have been minor changes to the protocol:

- January 2023: The introduction of an optional 6-month follow-up (self-report questionnaires only). Therefore, this was not offered to the first seven employers.
- August 2023: Two additional questions were added to the sleep diary to record naps and whether they worked the previous day, alongside qualitative feedback from employees and business leaders at the end of the trial.
- September 2024: Four questions added to record information on job title, biological sex, self-reported height and weight to calculate BMI and current diet.

### 2.7 Measures

The following sections describe the broad method and implementation model for each measure. There are four key data collection points: baseline, trial, end-of-trial, and 6-month follow-up. For all sections below, we collect identical data at the start and end of the trial, except for the “Work Patterns Questionnaire.” As shown in Figure 1, the “Weekly Goals and Questionnaires” are collected weekly from baseline week 1 through trial week 12 (i.e., over 14 weeks) and again at the 6-month follow-up. The “Workplace and Wellbeing Questionnaires” are collected once during baseline weeks 1 and 2, at the end of the trial (week 12), and at the 6-month follow-up. The “Work Patterns Questionnaire” is collected only in the last trial week (i.e., trial week 12). Data for the three opt-in remote measures, time-use diaries, sleep diaries, and sleep watches are collected during baseline weeks 1 and 2 and again at the end of the trial during trial weeks 10 and 11 (sleep watches and diaries), or week 11 (time-use diaries). For the three opt-in in-person visit measures at the University of Sussex, blood samples, MRI scans, and the body perception measure are collected in weeks 1 and 12 of the trial period. For working time reduction participants, the in-person measures are scheduled for the time they would otherwise have spent working during weeks 1 and 12.

### 2.8 Questionnaires

#### 2.8.1 Weekly performance: goal attainment

On the first day of each participant's working week, participants are emailed and asked to set five work-related goals for that week (based on Uhlig et al., 2023). Participants are told that these goals can be main tasks or responsibilities that they want to achieve by the end of their work week. Using Qualtrics (Provo, UT), participants then rate the extent to which they met their goals on their last working day of that week as part of the “Weekly Questionnaires” described below. Ratings are made on a five-point scale ranging from 1 (not at all) to 5 (goal met in its entirety), adapted from Harris et al. (2003) to measure goal attainment on a weekly basis. Participants are also given the option to respond with 0 if they did not set a goal. We do not record the goals set; instead, we ask participants to keep their own records to help evaluate whether they met them.

#### 2.8.2 Weekly questionnaire

On the last day of a participant's working week, they complete a battery of questionnaires on Qualtrics, including the weekly goal attainment evaluation from Section 2.8.1 and the following seven measures. Unless participants are taking leave for a full working week, they are still asked to complete the questionnaires, even if they work only 1 day.

**Positive and negative affect schedule (PANAS)**: A 20-item scale assessing positive and negative affect (Watson et al., 1988). Respondents rate 10 positive and 10 negative adjectives on a five-point scale based on how they have felt over the past week, from 1 (very slightly) to 5 (extremely). Positive and negative affect scores are summed separately, with higher scores indicating greater positive or negative affect.

**Maslach burnout inventory (MBI)**: A 22-item scale measuring burnout across three subscales: emotional exhaustion, depersonalization, and personal accomplishment (Maslach et al., 1996). Participants reflect on how much a statement applies to them over the past week. For example, “I feel emotionally drained by my work.” Responses use a seven-point scale from 1 (not at all) to 7 (completely). Scores on the emotional exhaustion scale range from 1 to 63, and on the depersonalization scale from 1 to 35, with higher scores indicating more severe burnout. Scores on the personal accomplishment scale range from 1 to 56, with lower scores indicating more severe burnout.

**Recovery experience questionnaire**: A four-item questionnaire assessing psychological detachment from work. Participants evaluate how much a statement applies to them over the past week (Sonnentag and Fritz, 2007). For example, “I forgot about work.” Responses are given on a five-point scale from 1 (I do not agree at all) to 5 (I fully agree), with higher scores indicating increased psychological detachment from work.

**Work engagement questionnaire**: A nine-item questionnaire evaluating how participants feel at work (Schaufeli et al., 2006). Participants rate the extent to which a statement reflects how they feel about their work in the past week on a six-point scale from 0 (never) to 6 (always). For example, “At my work, I feel bursting with energy,” with higher scores indicating a more positive work-related state of fulfillment.

**Task performance questionnaire**: A seven-item subscale of employee performance on in-role behavior from the job satisfaction and organization commitment questionnaire (Williams and Anderson, 1991). Ratings are given on a five-point scale, from 1 (strongly agree) to 5 (strongly disagree), reflecting on the past week at work. Although the original questionnaire's scale ranges from 1 (strongly disagree) to 5 (strongly agree), we reversed the response options to encourage participants to actively think about their response, by changing the format of the scale between questionnaires. The first five items are reverse coded, so that higher scores indicate better task performance. The seven statements selected are:

1. Adequately completed my assigned duties.
2. Fulfilled responsibilities specified in my job description.
3. Performed tasks that are expected of me.
4. Met the formal performance requirements of the job.
5. Engaged in activities that will directly affect my performance evaluation.
6. Neglected aspects of the job that I am expected to perform.
7. Failed to perform my expected duties.

**Pittsburgh sleep quality index (PSQI)**: A single modified question from the 19-item PSQI to measure sleep quality over the last week, rather than a month: “During the past *week*, how would you rate your sleep quality overall?” on a four-point scale from 0 (very good) to 3 (very bad). A higher score indicates poorer sleep quality (Buysse et al., 1989).

**Adherence check**: To assess whether participants are following their intended working time, participants in the working time reduction group are asked whether they worked on their fifth free day, “On your 4-day week work-free day, did you do any work? This includes engaging in work communications, such as email or phone calls.” with four possible responses: not at all, some of the day, most of the day, and all of the day. If participants indicate they worked on their work-free day, we ask an open-ended follow-up question as to why. Those who followed a 9-day fortnight are presented with the same two questions to check whether they worked, with “4-day week work-free day” replaced by “9-day fortnight work-free day.”

Participants in the control group have an alternative phrasing for this question, given that they do not have a fifth free day: “*Over the course of the past working week, please indicate the extent to which you were undertaking work during your contracted hours each day (including work communications such as emails or phone calls). Please select the option that best reflects your engagement for the average working day this week*.” Responses use the same 1–4 scale as for working time reduction participants, but with some additional detail on the amount of work undertaken: (1) Not at all [Undertook no work during contracted hours], (2) Some of the day [Worked for less than half of contracted hours], (3) Most of the day [Worked for more than half of contracted hours], and (4) All of the day [Fully engaged in work for all contracted hours].

**Annual leave check**: Initially, we only checked leave taken during the trial in the “Work Patterns Questionnaire” at the end of the trial (see Section 2.8.5); however, participants reported difficulty remembering all leave taken (e.g., half days for medical appointments). Therefore, in July 2024 we introduced an additional question to the “Weekly Questionnaire” to check any leave taken during the working week just experienced. We do not record the reason, only the time taken.

#### 2.8.3 Wellbeing questionnaire

During the baseline period, end-of-trial period, and again at a 6-month follow-up, participants are asked to complete the following six questionnaires and report their BMI. In addition, at baseline, participants complete seven demographic, lifestyle, and socioeconomic questions and, if participating in an MRI scan, report any medications they are taking.

**Depression anxiety stress scales (DASS)**: A 21-item questionnaire that includes three subscales: (1) depression, (2) anxiety, and (3) stress. Participants reflect on how much a statement applied to them over the last week. For example, “I couldn't seem to experience any positive feeling at all.” Responses are on a four-point scale from 0 (never) to 3 (almost always). The scores for each subscale range from 0 to 42, with higher scores indicating greater severity of depression, anxiety, or stress symptoms (Henry and Crawford, 2005).

**Wellbeing (WHO)**: A five-item questionnaire to measure mental wellbeing. Participants report how much a statement applied to them over the last 2 weeks. For example, “I have felt cheerful and in good spirits.” Responses are given on a six-point scale from 5 (all the time) to 0 (at no time). Scores range from 0 to 25, with 0 representing the lowest mental wellbeing and 25 the highest (World Health Organization, 1998).

**Body perception questionnaire (BPQ)—awareness subscale**: The first 45 items, forming the first awareness subscale, from the full 122-item Body Perception Questionnaire (Porges, 1993). Participants reflect on how aware they are of their body processes. For example, “During most situations I am aware of swallowing frequently.” Responses use a five-point scale from 1 (never) to 5 (always). Higher scores indicate greater awareness of internal bodily sensations.

**International physical activity questionnaire-short form (IPAQ)**: A 12-item questionnaire of an individual's physical activity over the previous 7 days, focusing on time spent on four activities: vigorous activity, moderate activity, walking, and sitting. For example, “During the last 7 days, on how many days did you do vigorous physical activities like heavy lifting, digging, aerobics, or fast bicycling?”. Answers are converted to METs, which are the energy requirements for a given task relative to resting energy expenditure, expressed as MET-minutes/week (i.e., a metabolic equivalent of task expressed in minutes per week). A MET value is assigned to the three active categories (i.e., vigorous activity, moderate activity and walking) using the formula “duration × frequency per week × MET intensity per activity type.” These scores are then also summed to create a total physical score for all activities per week (Craig et al., 2003). The higher the score, the more physically active a person is. A summary score is also given for time spent sitting.

Scores can also be further categorized into low, moderate, or high physical activity levels (Craig et al., 2003). Participants are classified as high if they engage in vigorous activity at least 3 days per week, with a minimum of 1,500 MET-minutes/week, or if they achieve at least 3,000 MET-minutes/week and engage in some form of physical activity every day of the week. There are three potential criteria to be classified as moderate: spending at least 20 min of vigorous activity on three or more days; or across five or more days completing at least 30 min of moderate activity or walking each day; or finally, if people achieve a minimum of at least 600 MET-minutes/week by taking part in any physical activity in five or more days. Finally, people are classified as having low activity levels if they do not meet the criteria for moderate or high.

**Food frequency questionnaire (FFQ)**: Participants indicate how often they consume portions of a range of common foods and drinks on a typical week in the last month (Cleghorn et al., 2016). This includes fruits, vegetables, meat, fish, types of milk and alcohol. Responses depend on the specific foods, but provide a “rarely/never” category that increases to “5+a day” or “7+a week.” The frequency of intake of fruit, vegetables, oily fish, non-milk extrinsic sugars, and fats is used to derive a diet quality score. These five components are considered to be important indicators of a healthy diet (World Health Organization, 2003). To calculate the diet quality score the number of reported servings consumed for each of the five components are compared against the UK dietary recommendations, resulting in a score of 1–3 per food component, where 3 represents meeting the recommendations (see Cleghorn et al., 2016). Therefore a minimum score is five and a maximum is 15, whereby higher scores indicate greater diet quality (i.e., indicators of a healthy diet).

**Pittsburgh sleep quality index (PSQI)**: A 19-item questionnaire which generates seven component scores reflecting usual sleeping habits during the past month: (1) subjective sleep quality, (2) sleep latency, (3) sleep duration, (4) sleep efficiency, (5) sleep disturbance, (6) use of sleeping medication, and (7) daytime dysfunction (Buysse et al., 1989). The required responses vary by question, for example “During the past month, how would you rate your sleep quality overall?” with four possible responses (i.e., very good, fairly good, fairly bad, very bad) to “During the past month, what time have you usually gone to bed at night?” with an open response to record the time. The seven component scores are derived on a four-point scale from 0 (no difficulty) to 3 (severe difficulty), with equal weighting, which are then summed into a global score ranging from 0 to 21. A higher score indicates poorer sleep quality.

**Body mass index (BMI):** As an indicator of overall body size relative to a person's height, participants are asked to self-report their height in cm and weight in kg, both to one decimal place. These are used to calculate BMI (weight ÷ height^2^). In general, a BMI of 25 or higher indicates that a person is overweight, while a score under 18.5 is classified as underweight.

**Demographic, lifestyle, and socioeconomic status:** At baseline, participants are asked seven questions, including three demographic questions on date of birth, gender (four options: male, female, non-binary or not listed, and prefer not to answer) and biological sex (four options: male, female, prefer not to answer, and I wish to give more information with an open text box to respond).

To gauge current socioeconomic status, three questions including current postcode, average total household income before tax (seven responses: < £18,000, £18,000–£30,999, £31,000–£51,999, £52,000–£100,000, and > £100,000, don't know and prefer not to answer) and current level of qualifications (eight responses: college or university degree, A-levels/AS levels or equivalent, O-levels/GCSE or equivalent, CSEs or equivalent, NVQ or HND or HNC or equivalent, other professional qualifications e.g., nursing, teaching, none of the above, prefer not to answer). Finally, there is one lifestyle question: “*In a typical day, how many hours do you spend driving?*” with four responses: an open text box and three further options: less than an hour a day, do not know, and prefer not to answer. Participants can opt not to answer any question.

**MRI medication:** If participants are taking part in an MRI session, we record at baseline and at the end of the trial any medications currently taken, and doses, to control for in MRI analyses.

#### 2.8.4 Working life questionnaire

During the baseline period, end-of-trial period, and again at a 6-month follow-up, participants are asked to complete the following nine questionnaires and a single question on their current job title.

**Current job title**: As roles within companies are diverse, in September 2024 we added an open-ended question to ask their current job title.

**Boundary preference**: A nine-item questionnaire to measure the level of separation a person has between work and personal roles (Kossek et al., 2006). Participants rate their agreement with the nine statements on a five-point scale from 1 (strongly agree) to 5 (strongly disagree). For example, “*I only take care of personal needs at work when I am ‘on break' or during my lunch hour*.” Low scores indicate greater separation between work and personal roles, whereas high scores indicate greater integration between work and personal roles.

**Job control**: Six modified questions inspired by a nine-item questionnaire to measure autonomy at work, including when, what, and how participants carry out work tasks (Breaugh, 1985). Participants rate how much each statement applies to them in their current job on a five-point scale (i.e., never, seldom, sometimes, often, and very often); however, the original questionnaire used a seven-point scale. The higher the score, the more autonomy they have in their job. The six questions include:

1. Can you choose how you do your job?
2. Can you decide when to do particular work activities?
3. Can you control the sequencing of your work activities?
4. Are you allowed to decide how you get your job done?
5. Do you have some control over what you are supposed to accomplish?
6. Are you able to modify your job objectives?

**Job support**: A four-item questionnaire to measure support at work. Participants report how well a statement applies to them when considering their current job role (Daniels, 2000). For example, “*Can you confide in other people at work?*”. Responses are given on a five-point scale from 1 (never) to 5 (very often), with higher scores indicating greater support at work.

**Job satisfaction**: A three-item questionnaire to measure satisfaction at work (Brasher and Chen, 1999; Cammann et al., 1983). Participants report how much a statement applies to them while thinking about their current job. For example, “In general, I like working here.” In the version we chose to use, responses are given on a six-point scale from 1 (disagree very much) to 6 (agree very much); however, other versions have used five- or seven-point scales (Bowling and Hammond, 2008). The second item is reverse-coded. A final score is calculated from the average of the three items, with higher scores indicating greater job satisfaction.

**Life satisfaction**: A five-item questionnaire to measure satisfaction with life (Diener et al., 1985). Participants report how much a statement applies on a seven-point scale from 1 (strongly disagree) to 7 (strongly agree). For example, “*I am satisfied with my life*.” Higher scores indicate higher satisfaction with life. Scores can also be summed, with seven benchmark cut-offs provided: 5–9 (extremely dissatisfied), 10–14 (dissatisfied), 15–19 (slightly dissatisfied), 20 (neutral), 21–25 (slightly satisfied), 26–30 (satisfied), and 31–35 (extremely satisfied).

**Organizational commitment**: A twenty-four item questionnaire to measure three core components: affective, continuance, and normative commitment (Allen and Meyer, 1990; Meyer and Allen, 1991). Affective commitment (questions 1–8), refers to involvement in, identification with, and emotional attachment to the workplace. Continuance commitment (questions 9–16), covers commitment based on negative associations with leaving the workplace. Lastly, normative commitment (questions 17–24), measures feelings of moral obligation to stay at the workplace. Participants indicate how well a statement applies to them in their workplace on a seven-point scale from 1 (strongly disagree) to 7 (strongly agree). For example, “*I enjoy discussing my organization with people outside it*.” Higher scores indicate greater commitment to the workplace.

**Intention to quit**: Two items are drawn from one of seven subscales of a comprehensive workplace commitment questionnaire to assess employees' intentions to quit (Firth et al., 2004). While thinking about their current job, participants reflect on two statements using an adapted five-point scale from 1 (not at all) to 5 (very much). Higher scores indicate greater intention to quit, including “*How often do you think about leaving the job*?”, and “*How likely are you to look for a new job within the year next year?*”.

**Self-efficacy**: An eight-item questionnaire to assess self-efficacy in the workplace (Chen et al., 2001). Participants evaluate how much they agree or disagree with each statement on a five-point scale from 1 (strongly disagree) to 5 (strongly agree). For example, “*Even when things are tough, I can perform quite well*.” Higher scores indicate greater self-efficacy.

**Proactive personality scale**: A 10-item shortened questionnaire measuring proactive personality (Seibert et al., 1999), taken from Bateman and Crant's (1993) scale. We used the same questions but with adapted scale markers from Trifiletti (2009). Participants evaluate how much a statement applies to them on a seven-point scale from 1 (absolutely false) to 7 (absolutely true), with higher scores indicating a more proactive personality. For example, “*If I see something I don't like, I fix it*.”

#### 2.8.5 Work pattern questionnaire

At the end of the trial, participants are asked to complete eight questions confirming their work patterns over the 14-week period. These questions are identical to those taken on the international trials reported by Fan et al. (2025) and Schor et al. (2022). In addition, participants are asked five qualitative questions about their experiences during the trial. The exact wording for these questionnaires is provided in the supplementary section “*Work pattern questionnaire wording*,” and a brief summary of each measure is provided in text below.

**Working days**: Participants are asked to specify how many days per week they typically worked pre-trial, providing either an exact number or an estimated average if needed. This is measured using an open-answer text box, as well as eight possible response options ranging from 0 to 7 days. The same question is asked during the trial period to ascertain if or how this has changed from pre-trial.

**Working hours:** Participants are asked to specify how many hours per week they typically worked before the trial period, excluding time spent on breaks or commuting to/from work but including time spent catching up on work during evenings or weekends. Participants are asked to provide either an exact number or an estimated average if needed. The same question is asked during the trial period to ascertain if or how this has changed from pre-trial.

**Remote working**: Three related questions are asked to determine whether participants worked remotely and whether this has changed over the trial period. This includes whether participants work remotely, and how often this typically occurred both pre-trial and during the trial.

**Annual leave:** To check any leave taken during the trial period, participants are asked to provide all dates of leave taken during the 14 - week period. In addition, we have records of annual leave sent to us via email or in out-of-office messages.

**Experiences of the trial**: Three open-response questions specifically ask participants about their experiences during the trial. This includes reflection on whether the trial has felt successful for them as an individual and for their organization as a whole. Participants are also asked to reflect on perceived benefits or challenges of working with the University of Sussex.

#### 2.8.6 Business leader questionnaire

To confirm organization-level working hours and how the programme has gone for businesses, organizational leaders are sent a separate questionnaire at the end of the trial, consisting of 14 questions. The exact wording for these questionnaires is provided in the supplementary section “*Business leader questionnaire wording*,” and a brief summary of each measure is provided in text below. Controls are sent an adapted version of the questionnaire given to working time reduction trial companies.

**Staff and working hours**: Four questions cover information about the staff and their working hours in the organization. This includes the number of staff in the organization, the number taking part in the research programme (including the time management training, regardless of participation in the research), and the typical weekly working hours for full-time staff both prior to and during the trial period.

**Programme reflections:** Four questions are asked to understand the experiences of the business leader throughout the trial period. This includes reflection on perceived benefits of working with the University of Sussex, thoughts on how the programme has gone for their organization, whether they intend to retain any changes in working patterns implemented for the trial period for their organization, and whether they are interested in their organization participating in the 6-month follow-up phase.

**Experiences of the trial**: Nine open-response questions specifically ask business leaders about their experiences during the trial and with the research team. This includes reflection on useful insight gained from the programme, feedback on what could have been done better, surprises encountered during the programme, and perceived changes in working practices during the programme. In addition, three follow-up questions are included to ask for their permission to use quotes from these questions and data attributed to their organization in scientific reports or on promotional project materials.

### 2.9 Remote measures

In addition to the questionnaires, which all participants complete online, participants can opt in to two further remote measures: time-use diaries and sleep tracking using a sleep watch and diary. If participants are completing both the sleep-tracking and time-use dairies, these are sent in the post the week before their baseline or end-of-trial period begins; if only time-use dairies, these are sent via email or post depending on the preference of the participant.

#### 2.9.1 Time-use diaries

**Figure 2:**
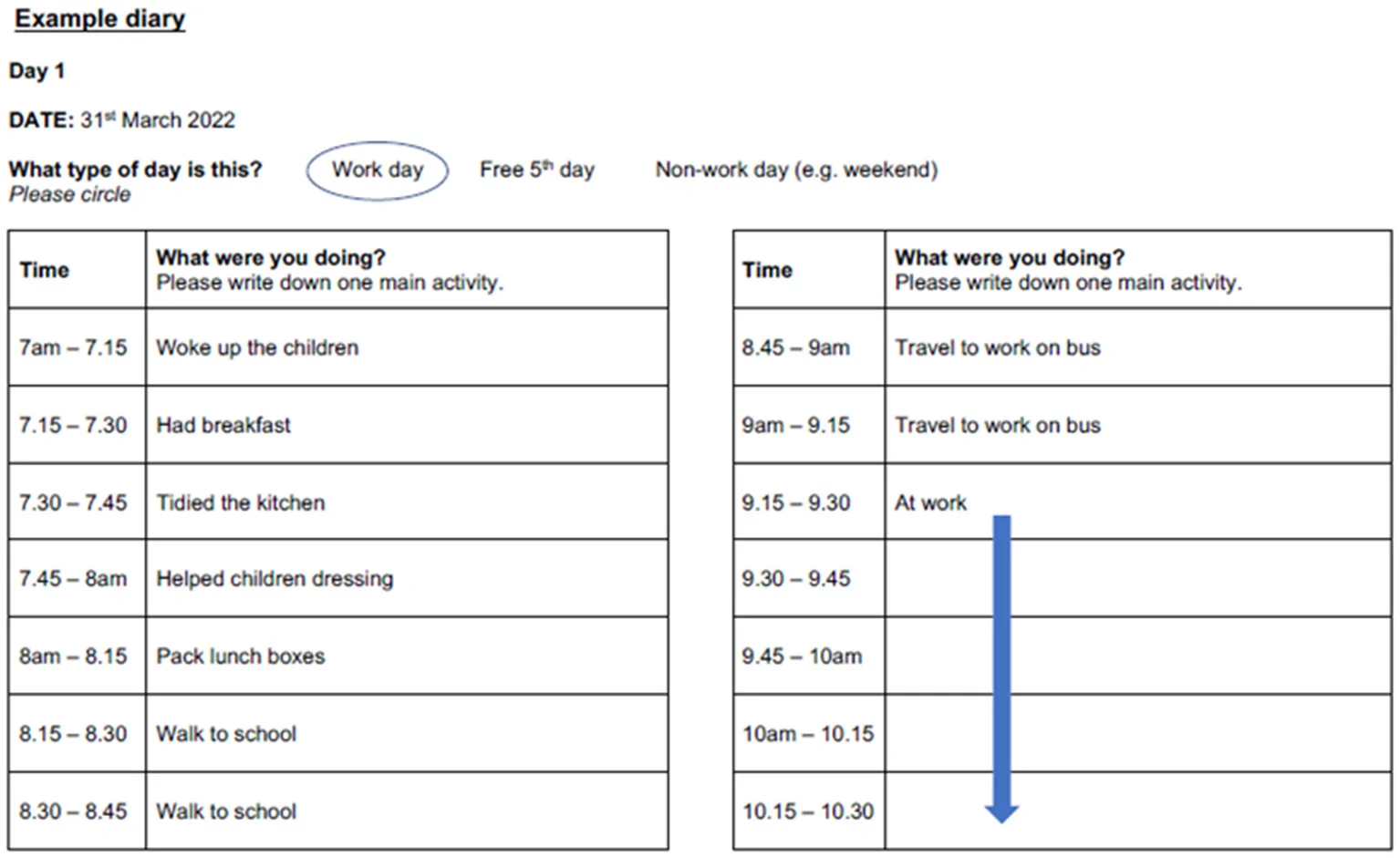
Example time-use diary section in time-use diary instructions.

Participants complete a time-use diary on 3 days during both the baseline and end-of-trial periods. At baseline, this includes 2 days at work, and 1 day they were not (e.g., a weekend day). At end-of-trial, participants in the working time reduction group complete diaries on one workday, the day they no longer work but previously did before the trial, and one other non-working day (e.g., a weekend). Participants with alternative working-time reduction arrangements (e.g., multiple half-days) are asked to complete the diary on one of their two half days, to ensure some time spent at work is captured. At end-of-trial, participants in the control group complete the time-use diaries as at baseline (i.e., two work days and one non-working day, e.g., a weekend day). Each diary includes two columns: one for the time, starting at 4 a.m. and covering a full 24-h period in 15-min intervals, and one for the main activity the participant is engaged in during each interval, adapted from the 2014–2015 UK Time Diary Study (Morris et al., 2016) to minimize participant burden. See Figure 2 for an example section of a time-use diary.

#### 2.9.2 Sleep watch and diaries

Participants can opt in to sleep-tracking over the course of the trial period. Participants wear a wrist-worn actigraph device (MotionWatch 8, CamNTech; https://www.camntech.com/motionwatch-8/) continuously throughout the 2 week baseline, and 2 week end-of-trial period (total of 14 days and nights in each period). Actigraphy records movement data, which is then translated into estimates of sleep-wake patterns using established algorithms which have been validated against gold-standard polysomnography (Elbaz et al., 2012), providing sleep onset, sleep latency, wake bouts, wake time, total sleep time, sleep efficiency, and other relevant metrics.

To validate the data collected using these watches, participants are asked to concurrently complete the self-reported “*Daily sleep diary*” from the Loughborough Clinical Sleep Research Unit (Morgan et al., 2007) each morning during the baseline and end-of-trial periods while wearing the watch. The original diary contains eight questions relating to sleep quality and duration. Two additional questions were added for the current project:

1. Did you have any naps yesterday? If so, what time, and for how long?
2. Were you at work yesterday? (Including working from home) *[Y* = *yes, normal day of work, X* = *a part day of work, N* = *no, was not at work]*

### 2.10 In-person visit

Measures administered in-person at the University of Sussex include a blood sample, an MRI brain scan, and interoception measures. Participants can opt in to any combination of these measures, which are completed at a single visit on both trial weeks 1 and 12.

#### 2.10.1 Blood sample

To assess serum concentrations of prototypical inflammatory markers, a 10 mL peripheral blood sample is collected via venipuncture into a BD Vacutainer^®^ serum tube. Samples are allowed to coagulate for 1 h after collection at room temperature, before centrifugation at 1,300 × g for 10 min at room temperature to separate the serum from the clotted material. Serum are stored at −80 °C until analysis.

#### 2.10.2 Interoception measures

Three measures assessing interoceptive accuracy and awareness are conducted in person using pulse oximetry on the index finger of the left hand (Nonin Xpod 3012LP with soft finger-mount). These three measures are summarized below (see Garfinkel et al., 2015; Koreki et al., 2020, 2022 for further details) and are collected at baseline and at end of trial.

*Heartbeat tracking task*. Participants report the number of perceived heartbeats at rest on six randomized trials of length 25, 30, 35, 40, 45 and 50 s. After each trial, participants rate their confidence in the accuracy of their response on a continuous visual analog scale from “total guess” to “complete confidence.” The VAS ranges from 0 to 1, but the scale numeric range and intervals are not displayed to participants. *Accuracy* is quantified by comparing the number of reported heartbeats to recorded pulses, according to: 1 – (|nbeatsrecorded – nbeatsreported|)/(nbeatsrecorded + nbeatsreported)/2. Scores are averaged across the six trials to produce a single accuracy and confidence value for each participant. *Awareness* is calculated by the Pearson correlation between accuracy and confidence values across trials.

*Time tracking task*. Operates exactly as the heartbeat tracking task, except that participants count number of seconds rather than number of heartbeats. This task is taken to check for non-interoceptive strategies. Specifically, there is evidence that performance on the heartbeat tracking task may be informed by non-interoceptive factors, notably knowledge concerning own heart rate and time estimation ability (Koreki et al., 2020).

*Heartbeat discrimination task*. On each trial, 10 auditory tones (440 Hz, 100 ms) are presented either synchronously or asynchronously (delayed) relative to heartbeats. Synchronous tones are triggered at the rising edge of the pulse pressure wave. Asynchronous tones are presented after a 300 ms delay. After each trial, participants judge if tones were synchronous or asynchronous relative to their heartbeats, then rate their confidence in their accuracy on a continuous VAS, as in the *heartbeat tracking task*. Participants complete 60 trials *Accuracy* is quantified by the number of correct trials divided by total trials. *Awareness* is calculated as the area under the receiver operating characteristic (ROC) curve, relating accuracy and confidence scores across trials.

*Trait interoceptive prediction error*. Interoceptive accuracy on the tracking and discrimination tasks, and Body Perception Questionnaire scores, are converted to standardized *z*-values. TIPE values are calculated for each participant according to the difference between *z*-scored Body Perception Questionnaire and *z*-scored accuracy (once for tracking, once for discrimination; Garfinkel et al., 2016).

#### 2.10.3 MRI scan

For participants who volunteer for the magnetic resonance imaging (MRI) brain scan, MRI compatibility is determined through screening questions for contraindications, including implanted medical devices and recent surgeries. Eligible participants then complete a brain MRI scan at baseline and again at end-of-trial. Scanning sequences are from the Human Connectome Project Development and Aging protocol (HCP D/A; Harms et al., 2018). These protocols are commonly used within human neuroimaging, and provide high temporal and structural resolution (Glasser et al., 2016). Specific scans run include functional MRI (fMRI; task-based and resting-state), diffusion-weighted imaging, and T1- and T2-weighted structural scans. Details of MRI sequences and task paradigms are provided below.

##### 2.10.3.1 Structural scans—T1 & T2

A high-resolution T1-weighted volume is acquired at baseline using an MPRAGE sequence with volumetric navigators (vNav) for prospective motion correction: TR = 2,500 ms, TE = 1.81 ms, TI = 1,000 ms, flip angle = 8 °, in-plane GRAPPA acceleration factor = 2, 0.8 mm isotropic voxels, matrix = 320 × 298, 208 sagittal slices, duration = 8 min 22 s. Four echoes are acquired and combined as a root-mean-square (RMS) image to enhance SNR. A matched T2-weighted volume is acquired at end of trial using a SPACE sequence with vNavs: TR = 3,200 ms, TE = 564 ms, variable flip angle (peak 120 °), in-plane GRAPPA acceleration factor = 2, 0.8 mm isotropic voxels, matrix = 320 × 300, duration = 6 min 35 s.

##### 2.10.3.2 FMRI parameters

Functional data are acquired using a multiband gradient-echo EPI sequence: TR = 800 ms, TE = 37 ms, flip angle = 52 °, multiband acceleration factor = 8, 2 mm isotropic voxels, matrix = 104 × 104, effective echo spacing = 0.58 ms, total readout time = 59.7 ms. Eight BOLD runs are acquired per participant. Two stop signal and two interoception task runs are collected (one of each per session, with phase encoding in the posterior-anterior direction). Four resting-state runs are collected (two per session) with alternating phase encoding (anterior-posterior and posterior-anterior) to enable distortion correction. Spin-echo field map pairs with opposing phase-encoding directions (AP and PA) are acquired adjacent to each set of functional runs to support EPI distortion correction: TR = 8,000 ms, TE = 66 ms, flip = 90 °, identical EPI geometry to the BOLD runs. A single band reference image is collected prior to the start of each fMRI run.

##### 2.10.3.3 FMRI—stop signal task

**Figure 3:**
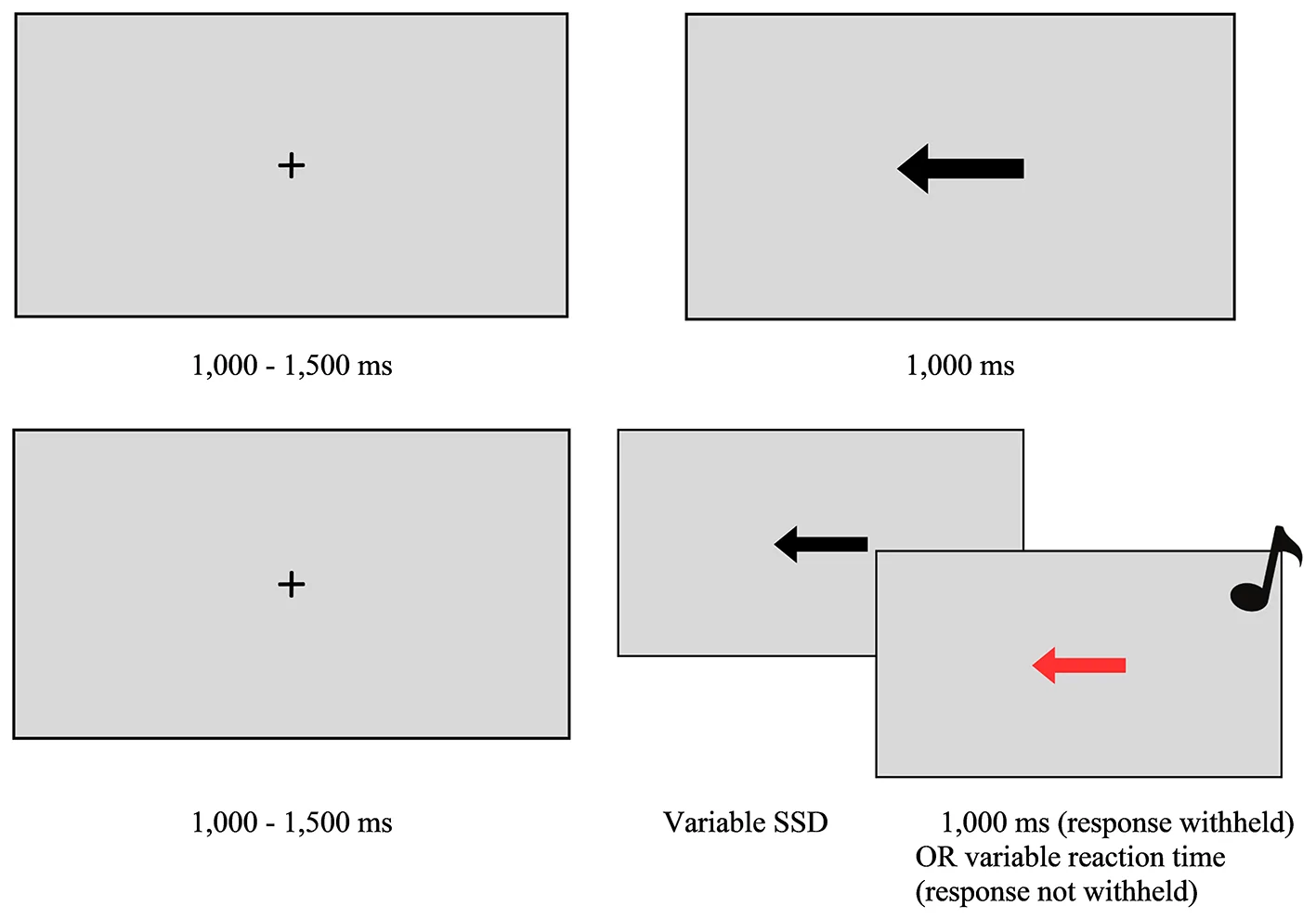
Procedure for the functional magnetic resonance imaging stop signal task. On “go” trials, a black arrow pointing left or right is presented for 1,000 ms, and participants indicate arrow direction using a button response. On “stop” trials, a “go” black arrow is initially presented and then replaced by a red arrow alongside an audio tone after a variable stop signal delay. Trials are preceded by a fixation cross of variable duration.

The stop-signal task consists of a single run, with a possible maximum of 750 volumes over 10 min. 240 trials are presented in total, including 180 “go” and 60 “stop,” presented in a fully randomized order. In each trial (Figure 3), participants are presented with an arrow pointing either left or right. Participants are encouraged to respond by pressing a button as quickly and as accurately as possible using a button box in their right hand (left arrow = index finger, right arrow = middle finger). On “stop” trials, after a short variable delay, the presented arrow turns red and an audio cue (1,000 Hz, 100 ms) sounds, indicating that the participant should withhold the button press.

Go trials have a set duration of 1,000 ms. For the first stop trial, a stop signal delay (SSD) of 250 ms is used. Over subsequent stop trials, this value adaptively responds to a participant's performance, such that SSD decreases by 50 ms if the previous response was correctly withheld, and increases by 50 ms if not. This adaptive design helps to ensure that approximately 50% of stop trials are classified as successful and 50% as unsuccessful. This is important for subsequent calculation of the stop signal response time (SSRT), which rests on assumptions of the independent race model. This model assumes that a stop signal task constitutes an independent race between a “go runner” and a “stop runner” (Verbruggen et al., 2019). Participants employing deliberate strategies, in which anticipation of stop signals interferes with response time on go trials, can violate this assumption of independence, and undermine the adaptive modulation of SSD duration. As such, participants are explicitly instructed not to wait to see if the presented arrow turns red before responding, but to prioritize responding quickly and accurately to the arrow direction, and try to stop if the stop cue appears. Trials are separated by a fixation cross intertrial interval, with a variable duration of 1,000 ms (35%), 1,130 ms (30%), 1,250 ms (20%), 1,380 ms (10%), or 1,500 ms (5%). The design of this task was guided by best practice recommendations from Verbruggen et al. (2019).

##### 2.10.3.4 FMRI—interoception task

**Figure 4:**
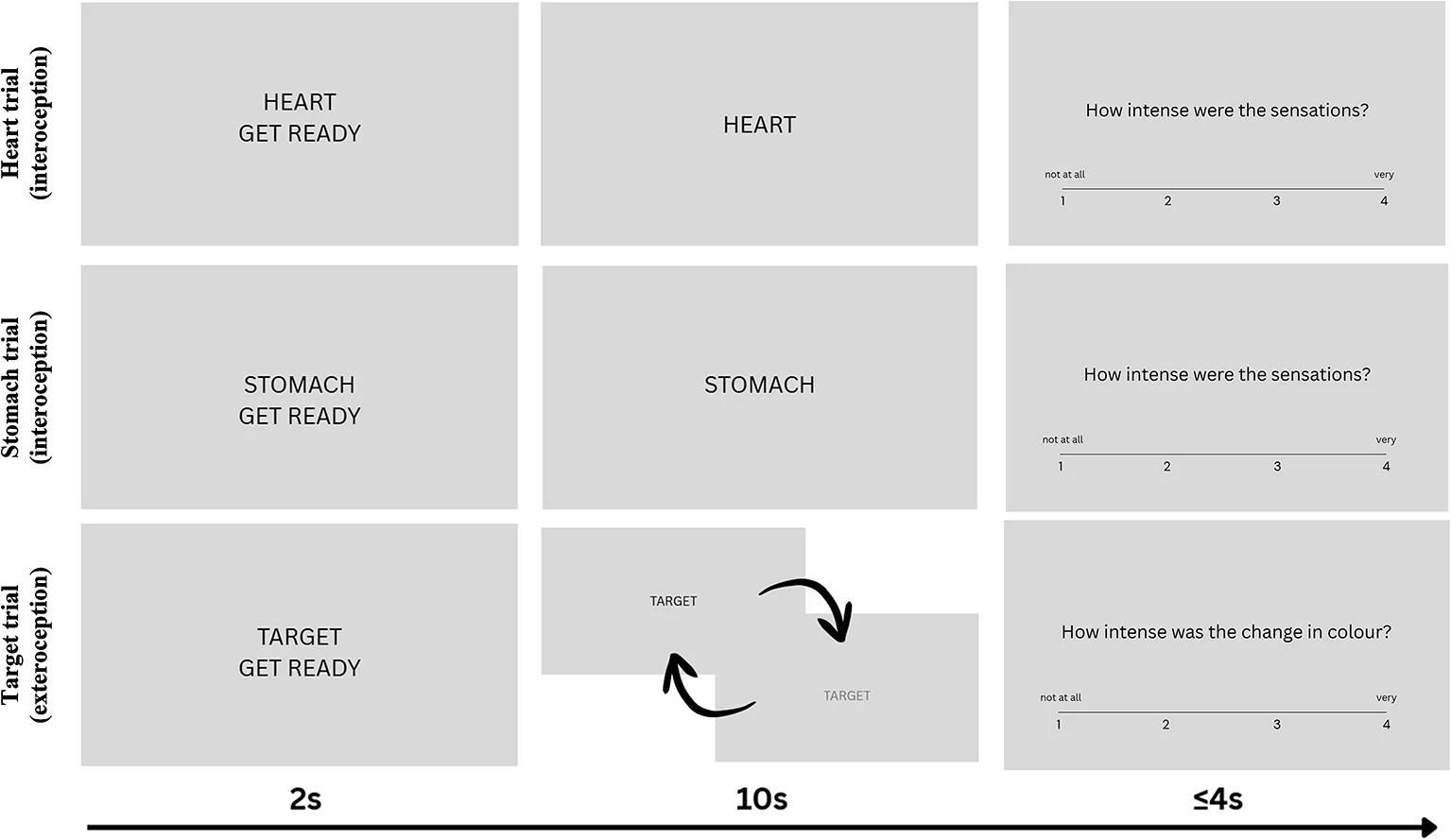
Procedure for functional magnetic resonance imaging interoception task. Stimuli are split according to trial type, including the interoceptive conditions of “heart” and “stomach” and the exteroceptive control condition of “target.” Stages of each trial are presented, including the “get ready” cue (2 s), the focus period (10 s), and the intensity rating period (up to 4 s).

The interoception task was adapted from the “Visceral interoceptive awareness task” described by Livermore et al. (2024). This task consists of one run, with a possible maximum of 1,125 volumes over 15 min. During this task, participants are presented with three trial types (Figure 4), two interoceptive and one exteroceptive:

- **Heart**: Participants are instructed to focus on the internal sensation of their heart.
- **Stomach**: Participants are instructed to focus on the internal sensation of their stomach.
- **Target**: Participants are instructed to attend to the word “Target,” which repeatedly changes color from black to a shade of gray with varying degrees of intensity, for a randomly selected duration between 0.7 and 1.1 s.

Following interoceptive and exteroceptive trials, participants rated the intensity of the internal sensations (HEART, STOMACH) or of the color change (TARGET), respectively, on a scale from 1 (“not at all”) to 4 (“very”).

Each session contains 48 trials (16 Heart, 16 Stomach, 16 Target). Each trial is preceded by a “get ready” instruction lasting 2 s. During each rating period, participants provide a response using a button box in their right hand (1 = index finger, 2 = middle finger, 3 = ring finger, 4 = little finger). Rating scales are presented (and responses logged) for a maximum of 4 s. If a response is provided before the end of this period, the prompt remains on screen for another 0.5 s before proceeding. The exact total length of the task is therefore variable across participants. Each rating period is followed by a fixation cross of a variable duration: 3 s (10.42%), 4 s (12.50%), 5 s (18.75%), 6 s (25.00%), 7 s (20.83%), 8 s (8.33%), 9 s (2.08%), 11 s (2.08%).

The task is identical to that used in (Livermore et al. 2024), except that intensity ratings are collected after every trial (rather than only on some trials in Livermore et al., 2024). Participants completed one of two fixed trial order sets in each session, with set order counterbalanced between participants.

##### 2.10.3.5 FMRI—resting-state

Resting-state fMRI is acquired in both AP and PA directions (2 runs total), with preceding spin echo field maps and identical BOLD acquisition parameters to the task-based parameters. Each run contains 565 volumes (7 min 53 s), for a combined total of 1,130 volumes (15 min 46 s). During these scans, participants are instructed to keep their eyes open and focus on a white fixation cross presented a black background.

##### 2.10.3.6 Diffusion-weighted imaging

Diffusion-weighted imaging is conducted across two runs (AP/PA), each with a scan time of 5 min 42 s per run (11 min 24 s total). Images are acquired using a multiband EPI sequence: TR = 3,230 ms, TE = 89.2 ms, flip angle = 78 °, multiband acceleration factor = 4, 1.5 mm isotropic voxels, matrix = 140 × 140, 92 axial slices, effective echo spacing = 0.69 ms, total readout time = 95.9 ms. Two diffusion shells are acquired (*b* = 1,500 and *b* = 3,000 s/mm^2^) with 47 and 46 directions per shell, respectively, per acquisition, plus 7 interleaved *b* = 0 volumes. Each shell direction set is acquired twice, once with anterior-posterior and once with posterior-anterior phase encoding, to enable distortion correction. A complementary 98-direction acquisition with the same shell structure is also collected in both phase-encoding directions, yielding a total of 197 unique diffusion-weighted directions per shell pair across the two acquisitions. Directions per shell are halved relative to the HCP standard sequence to reduce overall scan time.

Further details on all sequences are provided in the *Human Connectome Imaging protocols for the Lifespan Development and Aging* projects (Harms et al., 2018). All data are preprocessed using the HCP minimal preprocessing pipelines (Glasser et al., 2013) as implemented in QuNex 1.4.3 (Ji et al., 2023), with additional ICA-FIX denoising and MSMAll multi-modal areal-feature-based surface registration. Further details of which will be provided in the subsequent MRI-specific preregistration for this project.

For the duration of all MRI scan sequences, participants are equipped with a pulse oximeter (by default, on the index finger of their left hand). This allows for measurement and analysis of heart rate variability. This will enable deeper understanding of any changes observed on the interoception attention task, as changes in heart rate variability after working time reduction may be associated with changes in neural processes of interoceptive attention. Prior research suggests an association between interoceptive accuracy for heartbeat monitoring and heart rate variability (Lischke et al., 2021; Saito et al., 2025).

## 3 Statistical analysis

The primary objective of this research project is to understand how health, wellbeing, and work performance measures change following a reduction in working time, compared with a control group. The secondary objective will be to investigate whether individual differences, including variables such as sex, age, extent of working time reduction, and baseline score for a given measure, moderate these effects. In Sections 2.7–2.10, we summarized all data to be collected. The overall statistical analysis plans that apply across all data types are discussed below. Data-type specific statistical analysis plans will be expanded upon in greater detail in subsequent pre-registrations for each data type. We intend to first analyze each data type separately (e.g., diffusion MRI data, cytokines, time use diaries), and then integrate them using machine learning approaches to identify multimodal changes associated with the intervention.

### 3.1 Model assumptions and missing data

Prior to data analysis, model assumptions will be tested, including checks for normality, linearity, independence, multicollinearity, homoscedasticity, alongside extreme cases and outliers using techniques such as residual, Q-Q and scale-location plots, sample distribution, Cook's distance, and variance inflation factor (VIF). To ensure extreme cases do not drive effects, we will screen the data for outliers (e.g., using residuals, boxplots and histograms) and run models with and without these data points, confirming results with sensitivity checks, such as robust estimation where applicable. Where appropriate, we will use imputation to handle missing data.

### 3.2 Model design

This study uses a longitudinal mixed-effects design with a hierarchical structure. Repeated measures data are nested within employees, who are nested within companies, meaning observations are not independent and will be analyzed using multilevel methods. The design includes one between-subjects factor (group: intervention and control) and one within-subjects factor (time, which will vary per analysis with three datapoint options: baseline and end of trial; baseline, end of trial and 6-month follow-up; and the weekly questionnaires), with an interaction between time and group and corrections for multiple tests. When relevant, employee-level (e.g., age, household income) and company-level (e.g., industry, company size) covariates will be added to statistical models. Table 1 provides a list of all planned covariates. The independent variables will be fixed effects, with up to three random effects: time, employee, and organization. Covariates will be added to the model as fixed effects. This design enables us to determine whether changes are observed across time points for the working time reduction intervention group but not for controls.

**Table 1:** Planned individual-level and company-level covariates for inclusion in statistical models.

Category	Covariate	Type
Individual-level	Baseline value of dependent variable	Continuous
	Gender or sex	Categorical
	Household income	Categorical
	Education level	Categorical
	Age	Continuous
	Living area	Continuous
	Job title	Categorical
	Hours of working time reduction	Continuous
	Remote work status	Categorical
Company-level	Industry	Categorical
	Company size	Categorical
	Trial start month	Categorical
	Profit status	Categorical

The specific coding scheme used for categorical variables alongside any data specific covariates will be specified in future modality-specific preregistrations.

In line with other working time reduction trials, we will include all participants allocated to the intervention group in the primary analyses regardless of the actual extent of their working time reduction, unless the company average reduction of working hours is < 1 h per week. In which case, their data will be excluded from the main analyses. In secondary analyses, we will investigate whether intervention effects vary by the extent of working time reduction by adding this as a moderator in the mixed-effects models. The extent of working time reduction will be calculated as the difference between participants' self-reported weekly working hours before the trial and during the trial. Working time reduction will firstly be modeled as a continuous variable (hours reduced), but we will also explore potential non-linear relationships using categorical hourly brackets. Additional secondary analyses will investigate whether other individual characteristics moderate the intervention effects, such as sex, age, and baseline score for a given measure. Each potential moderator will be evaluated separately by including the relevant interaction terms in mixed-effects models.

Due to the range of data types and the number of variables, variable-specific hypotheses and model variations (i.e., specific moderators and additional covariates) will be addressed in multiple follow-up preregistrations on the Open Science Framework (OSF). These will provide further details on each data-type analysis within the OSF project repository.

### 3.3 Descriptive statistics and inference criteria

Descriptive statistics for each outcome variable, as well as basic demographic factors (e.g., age, sex, BMI, and socioeconomic status), and individual factors (included as covariates or moderators) will be reported. For continuous variables, this will include the mean, standard deviation, and range. For categorical variables, the raw number and percentage of the sample falling in each category will be reported.

To infer effects, the *F*-value, *p*-value (including 95% CI), and partial eta-squared effect size will be reported alongside standardized beta values and their 95% CI (i.e., parameter estimates) and intraclass correlations (ICC). An effect will be considered statistically significant if *p* < 0.05 and the corresponding 95% CI does not include zero.

## 4 Discussion

In this paper, we have outlined the protocol for the Sussex 4 Day Week study, in which a comprehensive dataset is collected across 14 weeks from participants before and after a meaningful reduction in working hours, alongside a control group with no change in working hours. Measures include self-reported work performance, wellbeing, sleep, and work patterns; time-use diaries; actigraphy and sleep diaries; MRI scans; blood samples; and laboratory measures assessing perception of internal body signals. In addition to providing essential context for future analyses using this dataset, we intend this protocol to serve as a model for researchers planning working time reduction interventions or occupational health studies.

A key contribution of this multi-modal study is its integration of subjective and objective measures. While recent working time reduction studies have primarily acquired self-report data, our protocol also includes diverse biomedical measures, including functional and diffusion MRI, inflammatory markers from blood samples, and actigraphy-based sleep assessments. These measures are less susceptible to expectancy or Hawthorne effects than self-report approaches. Although, given the diverse outcome variables being measured, it will largely not be possible to directly validate subjective measures using objective measures, with the exception of sleep, for which we will have both self-reported sleep duration and actigraphy watches and interoception, where we have the self-report body perception questionnaire and in-person measurements of interoceptive accuracy and awareness. However, the presence of any effects of working time reduction for the objective measures can be used as evidence that changes on any self-report measures are not solely the product of expectancy or Hawthorne effects. To our knowledge, this is the first working-time reduction study to examine changes in brain function, actigraphy-based sleep assessments and immune-related biomarkers, offering an opportunity to identify physiological mechanisms underlying changes in wellbeing and work performance.

Furthermore, sampling wellbeing and performance questionnaires each week will shed light on how individuals accumulate or conserve resources over time. COR theory purports that resource loss is more salient than resource gain for people (Hobfoll et al., 2018). Therefore, whilst our working time reduction intervention is likely to reduce resource loss (e.g., reduce burnout) and contribute to resource gains (e.g., improving wellbeing), we expect the greater impact to be on reducing resource loss-related outcomes, in keeping with the theory. This kind of dynamic tracking of resource loss and gain over time is considered important for better understanding how resource spirals emerge and how these impact work performance and wellbeing (Sonnentag and Meier, 2024). In the context of the Job Demands-Resources model (Bakker and Demerouti, 2007; Demerouti et al., 2001), we hypothesize that reduced working hours primarily function as a resource gain, leading to improvements in employee wellbeing and organizational outcomes. Although employees may experience pressure to complete the same workload within fewer days due to potential work intensification, we expect that the additional time off work will create such resource gain that wellbeing and performance will improve overall. This expectation is supported by previous research demonstrating wellbeing improvements following working time reduction (Fan et al., 2025). We also took stable measures of job and personal resources (e.g., job control, self-efficacy) to include as covariates in statistical models, given their established relationships with wellbeing and performance outcomes (Bakker et al., 2023).

The time-use diary data enables investigation into how activities undertaken during increased non-work time relate to employees' recovery experiences, and subsequently their wellbeing and performance (Sonnentag and Fritz, 2015; Vandoros et al., 2025). Sleep duration and quality may function as an additional key pathway of recovery through which resources are replenished (Sonnentag et al., 2008; Zijlstra and Sonnentag, 2006). The fMRI analysis will allow us to test the hypothesis that adaptive changes in brain function underlie benefits to both rest and motivation (Rae and Russell, 2025). While prior work has provided limited evidence of an association between working hours and brain function (Souter et al., 2026), the intervention design used in the current project will provide additional insight into mechanisms.

This protocol also addresses several limitations common in previous trials. Firstly, data collection spans multiple years (2022–2027), with employers initiating the research protocol at different times of the year. This rolling recruitment approach helps to reduce the influence of seasonal effects that may arise when data for all participants are collected on the same schedule. For instance, the non-profit 4 Day Week Global coordinated trials in which multiple employers were studied on the same calendar timeline, with a coordinated switch date to the new schedule, and cohort-level work reorganization support (Backmann et al., 2024; Fan et al., 2025; Gomes and Fontinha, 2024).

Secondly, control groups have not always been included in working time reduction studies (e.g., Lewis et al., 2023), or have used much smaller sample sizes than working time reduction groups (e.g., Backmann et al., 2024; Fan et al., 2025), or do not receive the same organizational training that enables isolation of changes due to working time reduction specifically (e.g., Fan et al., 2025). In contrast, our study includes a balanced control group of comparable size and sector composition, whereby control organizations complete the same protocols. This includes time management training, which allows stronger causal inference by isolating the specific effects of reduced working time from changes attributable to advice on productivity and time-management principles.

Nonetheless, this protocol has limitations. Our measures do not capture all factors that may influence responses to working-time reduction. For example, we did not collect information about participants' caregiving responsibilities or other non-work demands, which could moderate the benefits of reduced working hours. These choices reflect the need to minimize participant burden in an already intensive multi-modal protocol by focusing on measures most relevant to the biological mechanisms under investigation, acknowledging that other trials have focused more extensively on social and economic factors (e.g., Gomes and Fontinha, 2024; Lewis et al., 2023). In addition, due to in-person testing requirements, recruitment primarily relied on small to medium employers located near the research site who self-selected into either the working-time reduction or control group. This may limit generalizability to larger or geographically diverse organizations and may also introduce selection effects, as employers with more progressive work cultures were more likely to adopt working time reduction, with considerable variability in how organizations implemented working-time reduction (e.g., fixed vs. flexible days off). It must also be acknowledged that organizations from which participants are recruited self-select into either the working time reduction or control group. Given the need to carefully plan the nature and implementation of working time reduction, randomization of group assignment is not feasible. However, this does increase the likelihood of selection bias, with baseline characteristics in work culture and organizational structure impacting group membership, an issue inherent to working time reduction trials. Therefore, it will not be possible to entirely rule out expectancy effects, that observed benefits of working time reduction are driven by participants' expected improvement over the trial period. However, inclusion of baseline periods in both groups will allow us to observe whether such effects appear before the implementation of working time reduction. Finally, given the complex nature of this protocol and the involvement of the same researchers in both the data collection and analysis processes, it is not possible to entirely blind those performing the analysis to group membership (working time reduction vs control).

This protocol paper will be complemented by preregistered analysis plans for each data type described above, enabling transparent and reproducible analyses as findings emerge. Taken together, this protocol provides a rigorous and transparent foundation for examining the psychological and biological effects of reduced working time, offering comprehensive multimodal evidence which will support future theory, policy, and intervention design in the fields of working time reduction and occupational health.
