# Supplementary material for: Assessing health, wellbeing, and working life impacts of a 4-day week: a multimodal protocol
Source: Front Psychol. 2026 Aug 17;17:1894642. doi: 10.3389/fpsyg.2026.1894642 (PMC13527013; doi:10.3389/fpsyg.2026.1894642)
Supplement: Supplementary file 1 [file Table_1.docx]

**Assessing health, wellbeing, and working life impacts of a shorter working week: a multimodal protocol**

**Supplementary materials**

Armitage, R. M.*^a^, Souter, N. E.*^a^, Graftdijk, T. J. ^a^, McLaren, J. ^a^, Moore, S. A. ^a^, Kampoureli, C. ^a, b^, Racey, C. ^a, c^, Campbell-Meiklejohn, D. ^a^, Garfinkel, S. N. ^d^, Mullen, L. ^e^, Russell, E. ^f^, & Rae, C. L. ^a^

*joint first authors

# **Work patterns questionnaire wording**

Below we provide the full wording for questions included in the work patterns questionnaire.

**Working days**: To confirm how many days per week participants worked pre-trial, participants are asked, *“Before you started the [4-day-week trial /9-day fortnight trial /workplace wellbeing study], how many DAYS per week did you USUALLY work in your job? In the case of irregular working arrangements, please estimate an average, or describe your working arrangements in the text box*”. Alongside the optional open-answer text box, there are eight possible responses, ranging from 0 to 7 days. To understand how many days were worked during the trial, the same question was asked, but the first part of the question was changed to “*During the [4-day-week trial /9-day fortnight trial/workplace wellbeing study], …*”.

**Working hours:** To understand how many hours per week participants worked during the trial, participants are asked, “*During the [4-day-week trial /9-day fortnight trial/workplace wellbeing study], how many HOURS per week did you USUALLY work in your job? Exclude time spent on breaks and exclude time spent commuting to/from work. For example: 35 hour week = 9am-5pm x 5 days with a 1 hour lunch break each day. Include hours that you would usually spend working on your main job outside of your regular work schedule, such as catching up on working in the evenings or weekends. In the case of irregular working arrangements, please estimate an average, or describe your working arrangements in the text box*”. To understand how many hours were worked during the trial, the same question was asked, but the first part of the question was changed to “*During the [4-day-week trial /9-day fortnight trial /workplace wellbeing study], …*”.

**Remote working**: Three related questions were asked to determine whether participants worked remotely and whether this had changed over the trial period.

1. “*Do you work remotely at your job (i.e., work from home)?*” with three options: I am fully remote, I am sometimes remote, or I am never remote.
2. “*Before you started the [4-day-week trial /9-day fortnight trial/workplace wellbeing study], how many days per week did you usually work remotely?*” with eight options from 0 (N/A - none) to 7 days.
3. “*During the [4-day-week trial /9-day fortnight trial /workplace wellbeing study], how many days per week did you usually work remotely?*” with eight options from 0 (N/A - none) to 7 days.

**Annual leave:** To check any leave taken during the trial period, participants are asked to *“enter all of the annual leave dates taken over the last 14 weeks (i.e., the duration of your participation in this study). Please enter this in blocks of dates, using separate lines in the text box for each block*”. In addition, we have records of annual leave sent to us via email or in out-of-office messages.

**Experiences of the trial**: Three open-response questions specifically ask participants about their experiences during the trial, with two follow-up questions for their permission to use quotes in scientific reports or on promotional project materials (three options: no, yes with my name and business name, and yes but anonymously):

1. Self: “*Do you have any thoughts on how the [4-day-week trial /9-day fortnight trial/workplace wellbeing study] has gone for you, as an individual member of staff? For example, what have the benefits been? What hasn’t gone so well (if anything)?*”
2. Organisation: “*Do you have any thoughts on how the [4-day-week trial /9-day fortnight trial/workplace wellbeing study] has gone for you, for your organisation? For example, what have the benefits been? What hasn’t gone so well (if anything)?*”
3. University of Sussex: “*Do you have any thoughts on working with the University of Sussex as part of the trial? For example, what have the benefits been (for you, or for your organisation)? What wasn’t so useful?*”.

# **Business leader questionnaire wording**

**Staff and working hours**: Four questions cover information about the staff and their working hours in the organisation:

1. “*Please confirm the number of staff in your organisation*”.
2. “*How many of these staff in your organisation took part in this programme? (This may not be the same number who took part in Sussex research: here we want to know how many in your organisation received productivity training, regardless of whether they took part in the Sussex research tests)*”.
3. “*Please confirm what the original full-time working hours and working schedule were for your organisation, at the start of the study period” For example, 37.5 hour week; 9am-5.30pm x 5 days with a 1 hour lunch break, 37.5 hour week; worked across 5 days with flexible start and finish times, and a 1 hour lunch break or 40 hour week; worked flexibly across any days, and any start and finish times*”.
4. “*Please confirm whether full-time working hours and the working schedule for your organisation changed over the course of the study period, and if so, how they changed*”.

**Programme reflections:** Four questions are asked to understand the experiences of the business leader throughout the trial period, two open-response questions, one on working practices and a final on a 6-month follow-up.

1. “*Do you have any thoughts on how the University of Sussex programme has gone for you, as the organisation leader? For example, what have the benefits been? What hasn’t gone so well (if anything)?*”.
2. “*Do you have any thoughts on how the University of Sussex programme has gone for your organisation? For example, what have the benefits been? What hasn’t gone so well (if anything)?*”.
3. “*Do you intend to keep any change(s) in working practices that arose through the University programme going forwards?*” with eight response options:
   a. Yes, we are making the change(s) permanent.
   b. Yes, but we are not making the change(s) permanent yet.
   c. Maybe, we are continuing a trial phase for now.
   d. Maybe, we have paused to consider [please add any further info as to why].
   e. No, we are not intending to keep the changes, but plan to try something else. [please add any further info as to why, and what you plan to try next].
   f. No, we are not intending to keep the changes [please add any further info as to why].
   g. Not applicable, we did not make any changes to our working practices
   h. Other [please describe].
4. “*Are you interested in us conducting a 6-month follow-up with your organisation, to assess staff wellbeing and performance after a longer period of time?*” with three response options: ‘yes’, ‘maybe’, ‘can you get back in touch a couple of months before to check in?’, or ‘no, thank you’.

**Experiences of the trial**: Nine open-response questions specifically ask business leaders about their experiences during the trial and with the research team. In addition, three follow-up questions were included to ask for their permission to use quotes from these questions and data attributed to their organisation in scientific reports or on promotional project materials (five options: no, yes with my name and business name, yes with business name only, yes with name only, or yes but anonymously):

1. “*Do you have any thoughts on working with the University of Sussex as part of this project? For example, what have the benefits been (for you, or for your organisation)? What wasn’t so useful?*”.
2. “*What was the single most useful thing about working with us?*”.
3. “*What could we have improved or done differently?*”.
4. “*What were your initial reasons for engaging with this University of Sussex programme? Did you have any expectations going in?*”.
5. “*Where was Charlotte’s support most helpful over the course of this project?*”.
6. “*What challenges did you present Charlotte and her team with (especially any that may be unique to your organisation), and how did they help address them?*”.
7. “*How was the experience of the project itself? Did anything surprise you? What turned out to be easier or more difficult than expected?*”.
8. “*Have you noticed any changes in work practices over the course of this project? Where did you notice the biggest change?*”.
9. “*Do you have any advice for other business owners considering working with the University of Sussex programme team?*”.
